# SIRT1 activation attenuates α cell hyperplasia, hyperglucagonaemia and hyperglycaemia in STZ-diabetic mice

**DOI:** 10.1038/s41598-018-32351-z

**Published:** 2018-09-18

**Authors:** Yanling Zhang, Kerri Thai, Tianru Jin, Minna Woo, Richard E. Gilbert

**Affiliations:** 1 St. Michael’s Hospital, Keenan Research Centre, Li Ka Shing Knowledge Institute, Toronto, M5B 1W8 Canada; 2 Toronto General Hospital Research Institute (TGHRI), Toronto, ON M5G 2C4 Canada

## Abstract

The NAD^+^-dependent lysine deacetylase, Sirtuin 1 (SIRT1), plays a central role in metabolic regulation. With type 1 diabetes a disease that is characterised by metabolic dysregulation, we sought to assess the impact of SIRT1 activation in experimental, streptozotocin (STZ)-induced diabetes. CD1 mice with and without STZ-induced diabetes were randomized to receive the SIRT1 activating compound, SRT3025, or vehicle over 20 weeks. Vehicle treated STZ-CD1 mice developed severe hyperglycaemia with near-absent circulating insulin and widespread beta cell loss in association with hyperglucagonaemia and expanded islet alpha cell mass. Without affecting ß-cell mass or circulating insulin, diabetic mice that received SRT3025 had substantially improved glycaemic control with greatly reduced islet α cell mass and lower plasma glucagon concentrations. Consistent with reduced glucagon abundance, the diabetes-associated overexpression of key gluconeogenic enzymes, glucose-6-phosphatase and PEPCK were also lowered by SRT3025. Incubating cultured α cells with SRT3025 diminished their glucagon secretion and proliferative activity in association with a reduction in the α cell associated transcription factor, Aristaless Related Homeobox (Arx). By reducing the paradoxical increase in glucagon, SIRT1 activation may offer a new, α-cell centric approach to the treatment of type 1 diabetes.

## Introduction

Cell function is dependent on a constant supply of energy. While mammals and lower organisms have developed ways of storing energy, prolonged periods of low energy supply are almost inevitable and having the means to adapt to them have been evolutionarily critical. Among these adaptive mechanisms is SIRT1, a widely expressed NAD^+^-dependent lysine deacetylase that plays a central role in metabolic regulation when energy sources are limited. Included in its myriad of metabolic effects, SIRT1 activation augments fatty acid oxidation, ketogenesis and gluconeogenesis to provide endogenous sources of energy when food intake is limited or restricted^1^. Unsurprisingly, given its potential role in life- and health-span extension, pharmacological methods of SIRT1 activation have been vigorously pursued, initially with resveratrol and more recently with a range of more potent sirtuin-activating compounds or STACs^2,3^.

Although access to energy-rich nutrients is not restricted in diabetes pathophysiological changes such as glycosuria and unwarranted gluconeogenesis constitute sources of insensible energy loss. Previous studies that explored SIRT1 in diabetes have focussed on type 2 diabetes, showing that SIRT1 activation enhances ß-cell function, improves insulin sensitivity and increases ß cell mass to attenuate hyperglycaemia^4,5^. As such, its potential as a new therapeutic strategy in type 2 diabetes has been repeatedly suggested^6,7^. In contrast, the effects of SIRT1 activation in Type 1 diabetes, a disease that unlike type 2 diabetes is characterized by ß-cell loss and insulinopenia, has not been previously investigated outside of its potential role in autoimmunity^8^.

While cognisant of the complete or near-complete destruction of ß-cells in type 1 diabetes, we nevertheless postulated that SIRT1 may still exert its energy conserving effects in this setting but would need to do so through insulin-independent mechanisms. As such we focussed on glucagon, the glucose-elevating hormone that like insulin is also secreted by the Islets of Langerhans. Unlike insulin-producing ß cells that are destroyed in type 1 diabetes, not only are α cells preserved but become hyperplastic with inappropriately high circulating plasma glucagon that substantially contribute to the hyperglycaemia of this disorder^9–11^. Accordingly, we hypothesised that SIRT1 activation would reduce α cell hyperplasia and hyperglucagonaemia as a way of attenuating the insensible energy loss of hyperglycemia in type 1 diabetes.

## Results

### SIRT1 activation attenuates hyperglycaemia and hyperglucagonaemia

When compared with non-diabetic animals, mice with STZ-diabetes were all hyperglycaemic, had lower body weight, and increased urine output (Table 1). Among non-diabetic mice, body weight was lower in animals that had received SRT3025 despite similar food intake. In the diabetic groups, body weight and food intake were both lower in those mice that had received SRT3025.

**Table 1:** Animal characteristics.

N	non-DM + vehicle	non-DM + SRT3025	DM + vehicle	DM + SRT3025
	22	21	20	24
Body weight (g)	51.0 ± 1.6	46.8 ± 0.9^‡^	42.2 ± 0.9*	37.6 ± 0.7*^†^
Food intake (g/day)	5.1 ± 0.2	5.2 ± 0.3	11.2 ± 0.8*	6.4 ± 0.4^†^
Urine output (ml/day)	2.7 ± 0.3	2.2 ± 0.3	45.7 ± 3.2*	24.3 ± 1.9*^†^
Fasting glucose (mmol/l)	9.1 ± 0.7	8.7 ± 0.4	30.2 ± 3.1*	18.4 ± 4.3*^†^
Body temperature (°C)	36.7 ± 0.5	36.38 ± 0.3	33.4 ± 0.1*	36.3 ± 0.3^†^

*p < 0.05 vs. non-DM + vehicle and non-DM + SRT3025; ^†^p < 0.05 vs. DM + vehicle.

^‡^p < 0.05 vs. non-DM + vehicle.

**Figure 1:**
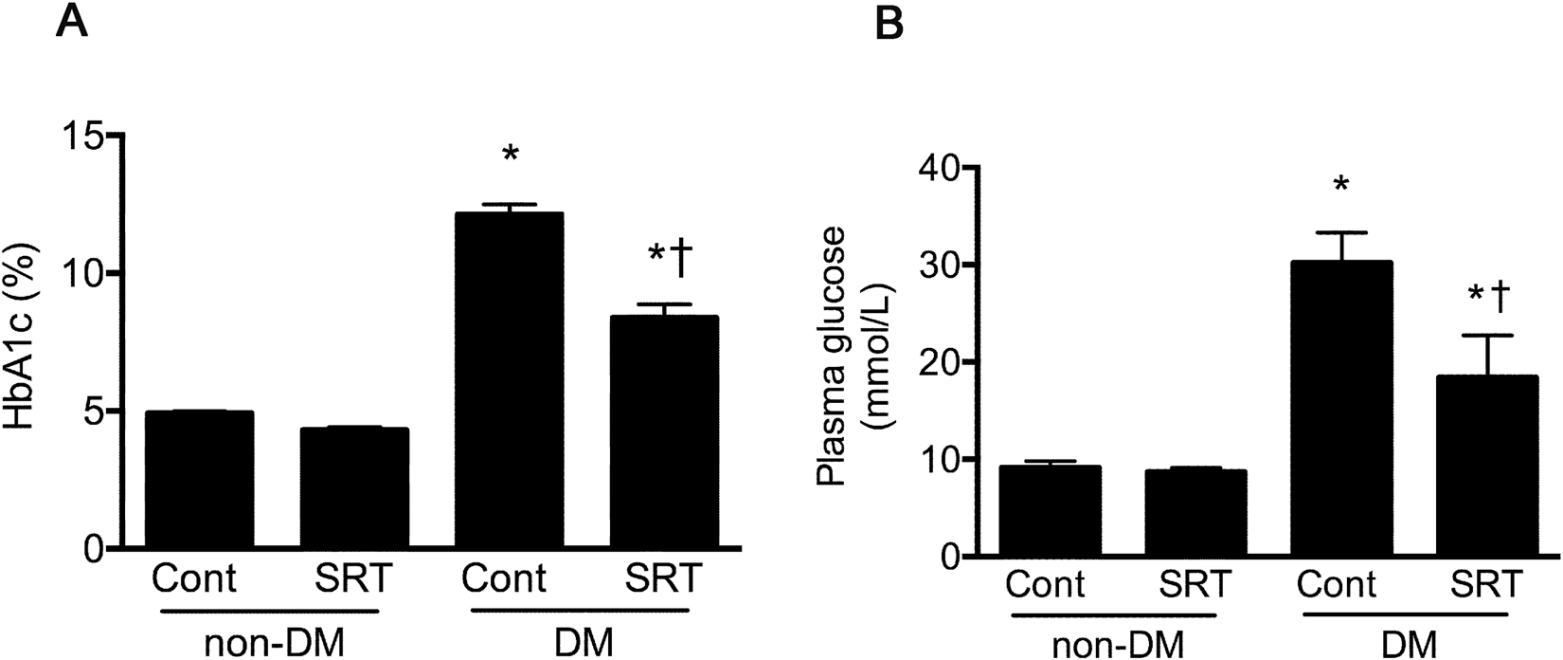
Haemoglobin A_1c_ and fasting plasma glucose in CD1 control and STZ-diabetic mice treated with vehicle or SRT3025. *p < 0.05 versus non-diabetic vehicle-treated mice, ^†^p < 0.05 versus vehicle-treated diabetic mice.

**Figure 2:**
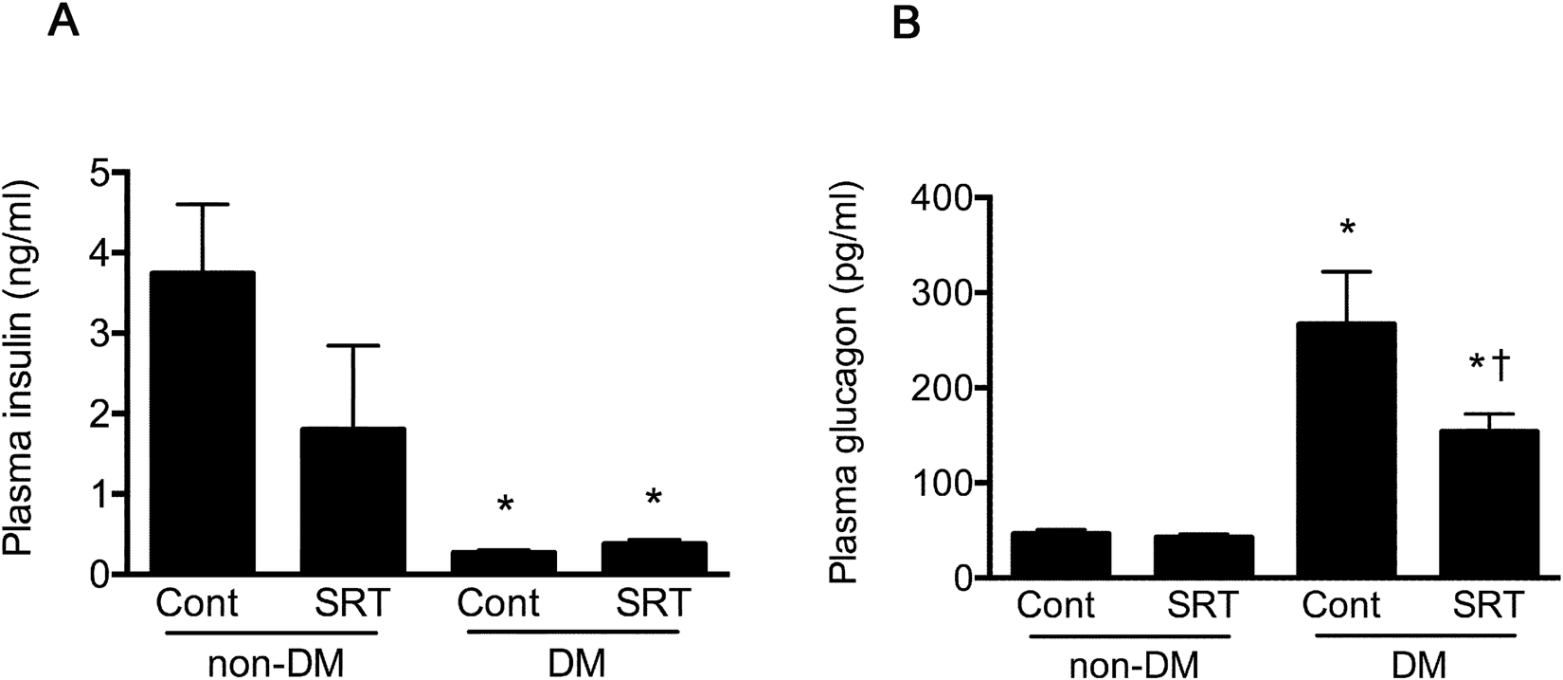
Plasma insulin and glucagon in CD1 control and STZ-diabetic mice treated with vehicle or SRT3025. *p < 0.05 versus non-diabetic vehicle-treated mice, ^†^p < 0.05 versus vehicle-treated diabetic mice.

Diabetic mice that had received SRT3025 had improved glycaemic control as manifested by lower HbA_1c_, fasting and non-fasting blood glucose concentrations (Fig. 1A,B). Fasting plasma insulin concentrations were below the limits of detection by ELISA in animals that had received STZ regardless of their assignment to receive SRT3025 or not and while detectable but very low in the non-fasting state, concentrations did not vary according to treatment assignment (Fig. 2A). Plasma glucagon was markedly elevated in untreated diabetic mice but was substantially reduced with SRT3025 (Fig. 2B).

**Figure 3:**
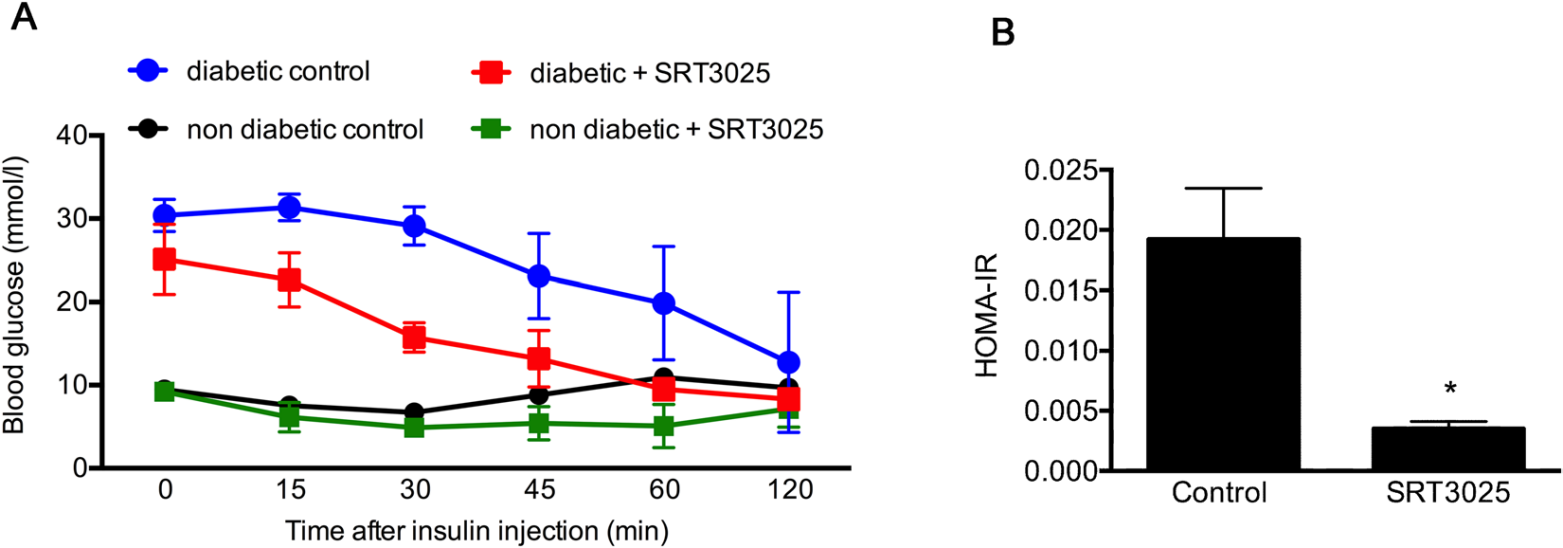
Insulin tolerance test (**A**) and HOMA-IR (**B**). When compared with untreated STZ-diabetic mice, those that had received SRT3025 had a greater response to exogenous insulin (p < 0.01). HOMA-IR that could only be assessed in non-diabetic mice was lower in animals that had received SRT3025. *p < 0.05.

In the non-diabetic setting plasma glucose, HbA_1c_, and glucagon concentrations did not differ according to treatment assignment. However, a trend towards a reduction in plasma insulin was evident in non-diabetic mice that had received SRT3025 (p = 0.06, Fig. 2A). The response to exogenous insulin was assessed by an insulin tolerance test (ITT). A time-dependent reduction in blood glucose was noted in all groups (p < 0.001, Fig. 3). Among diabetic animals, the decline in blood glucose was greater in those mice that had received SRT3025 (p < 0.01). No difference in time-dependent responses to insulin were evident between non-diabetic mice that had received either vehicle or SRT3025. However, the homeostatic model assessment of insulin resistance (HOMA-IR) was reduced in SRT3025-treated non-diabetic mice (Fig. 3). As a consequence of the extremely low concentration of insulin, HOMA-IR could not be assessed in STZ-diabetic mice.

Food consumption followed a similar pattern with increased intake in the setting of untreated diabetes that was tempered by the administration of SRT3025. When compared with non-diabetic control mice, body weight was lower in both diabetic groups but was also reduced in non-diabetic mice that had received SRT3025. Body temperature was lower in vehicle-treated diabetic mice but not in those that had received SRT3025. Table 1.

### SIRT1 activation diminishes islet α cell hyperplasia

**Figure 4:**
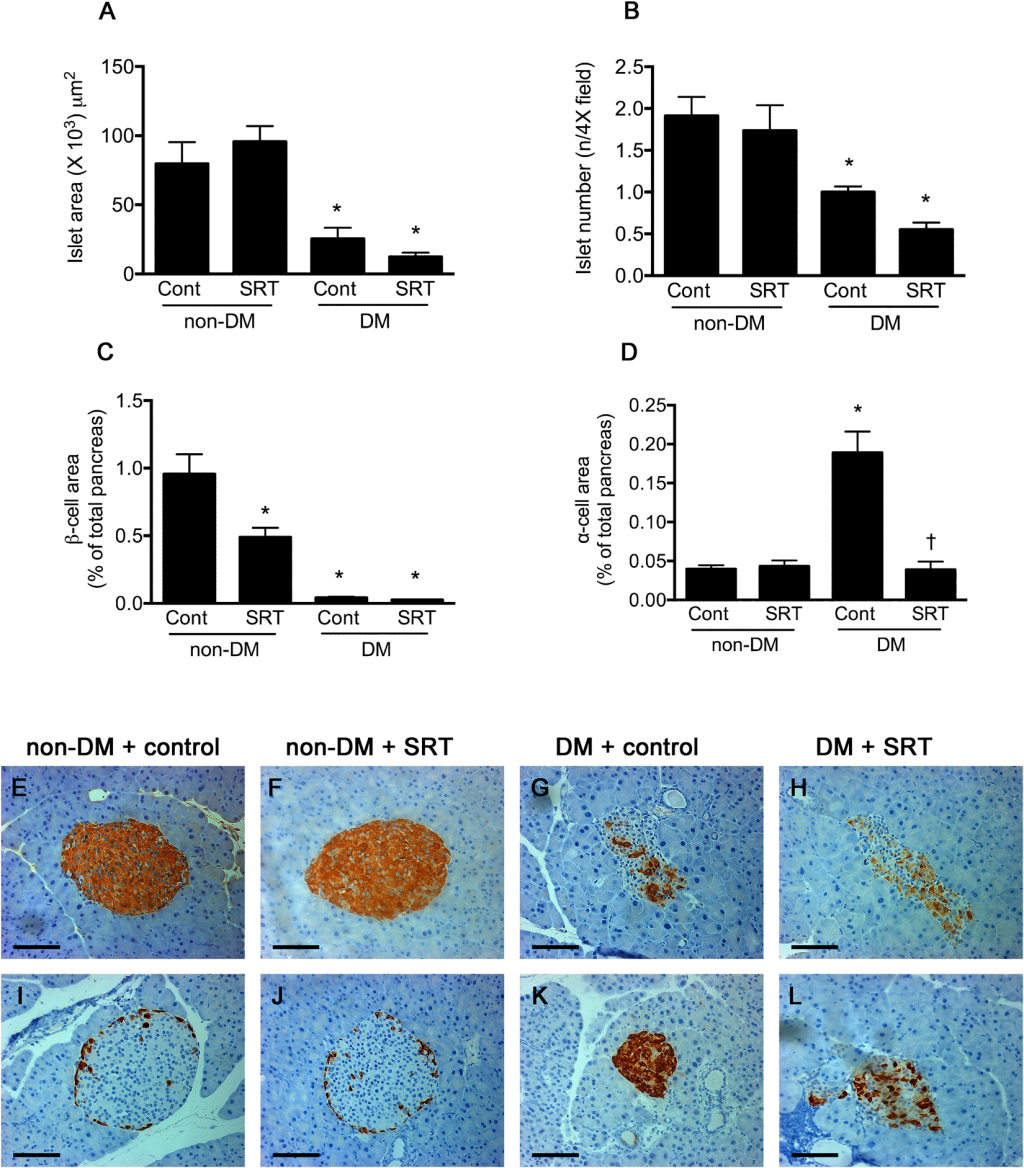
Islet morphology and immunohistochemistry. Pancreatic islets were fewer in number (**A**) and of smaller size (**B**) in STZ-diabetic mice but were unaffected by SRT3025. The abundance of ß-cells, expressed as the proportional area of insulin immunopositive cells in the pancreas, was lower in non-diabetic mice that had received SRT3025 than in untreated control with very few cells detected in STZ-diabetic mice, regardless of treatment assignment (**C**,**E–H**). The abundance of α-cells was increased in untreated STZ-diabetic mice when compared with diabetic mice that had received SRT3025. *p < 0.05 versus non-diabetic vehicle-treated mice, ^†^p < 0.05 versus vehicle-treated diabetic mice (**D**,**I**–**L**).

Islet size and number were lower in STZ-diabetic mice than in control animals regardless of treatment assignment (Fig. 4). Only sparse insulin immunolabelling of islets was detected in mice that had received STZ in contrast to its relative abundance in control animals. Immunostainable glucagon, on the other hand, was present in modest abundance in the periphery of islets from control mice in both groups in the classic mantle distribution in both SRT3025-treated and control non-diabetic groups. As expected, α cell mass was markedly increased in islets from control untreated diabetic mice (Fig. 4). Intriguingly, administration of SRT3025 led to a marked reduction in islet glucagon staining in diabetic animals even though this compound did not exhibit any effect on α cells in non-diabetic mice.

### SIRT1 activation diminishes gluconeogenic enzyme expression

**Figure 5:**
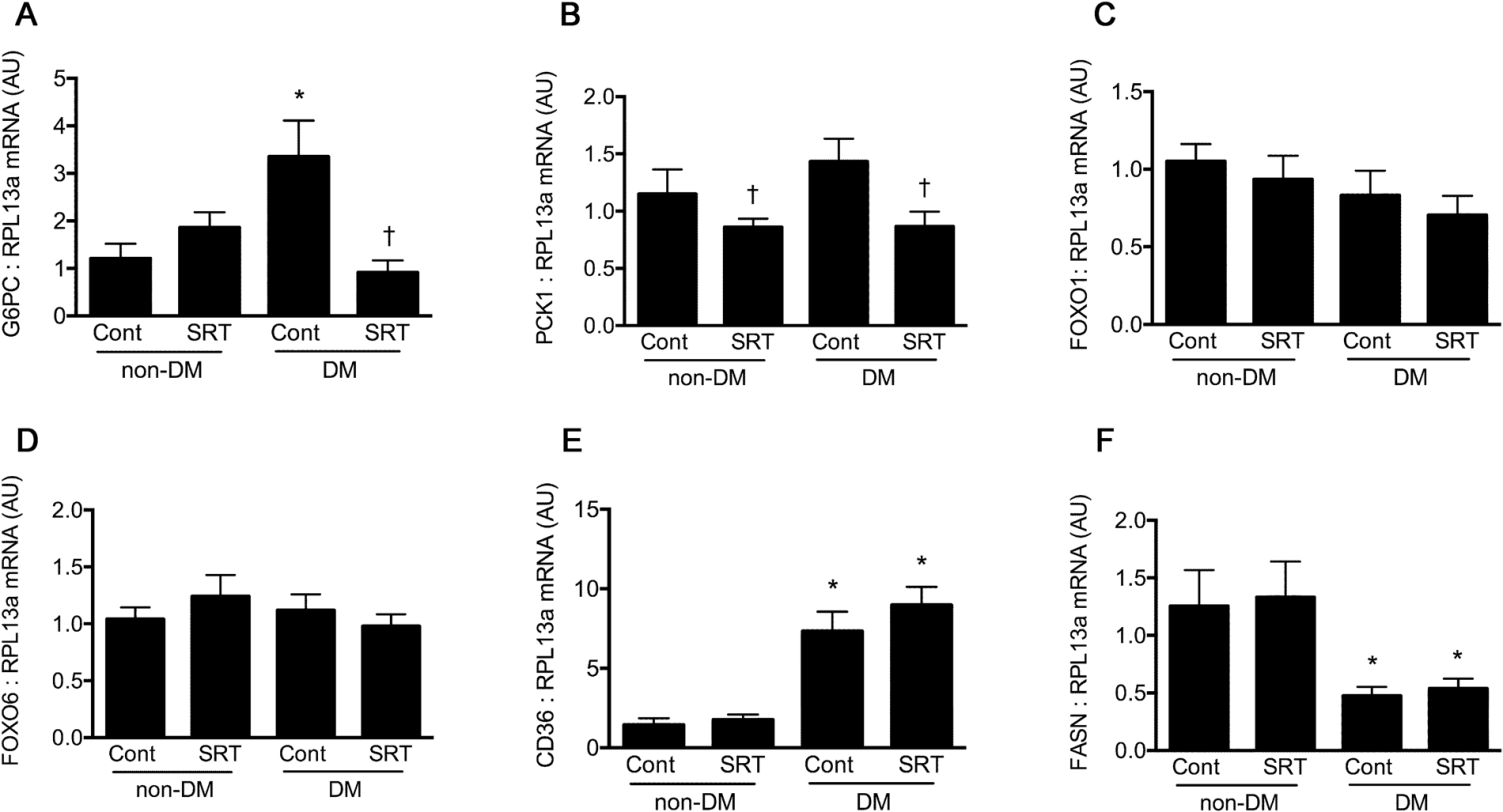
Liver gene expression of key gluoconeogenic enzymes: glucose 6-phosphatase (G6Pase), phosphoenolpyruvate carboxykinase (PCK1); insulin-suppressible transcription factors: forkhead box O1 (FoxO1) and FoxO6; and lipid-related factors: fatty acid synthase (FASN) and fatty acid translocase CD36 all expressed as arbitrary units (AU) relative to the housekeeping gene RPL13a with vehicle-treated non-diabetic mice arbitrarily assigned a value of 1. *p < 0.05 vs. control non-diabetic mice; ^†^p < 0.05 vs. untreated STZ-diabetic mice.

We next assessed the gluconeogenic effects of SIRT1 activation in the liver. We observed some differences in the enzymes that mediate hepatic glucose production consistent with the differences in the glucagon and α cell mass that were observed in response to SRT3025 (Fig. 5). G6Pase, a key enzyme in hepatic glucose production and release into the circulation, was increased in diabetes and reduced by SRT3025 in diabetic animals. PEPCK (PCK1), another key regulator of gluconeogenesis, was also reduced by SRT3025 in both diabetic and non-diabetic mice. Gene expression of other insulin-responsive metabolic enzymes, including transcription factors Forkhead Box O1 and O6 (FoxO1, 6) and the fatty acid transporter, CD36, and fatty acid synthase were both modulated by diabetes but were unaffected by SRT3025 (Fig. 5).

### SIRT1 activation suppresses glucagon transcription and α cell proliferation

**Figure 6:**
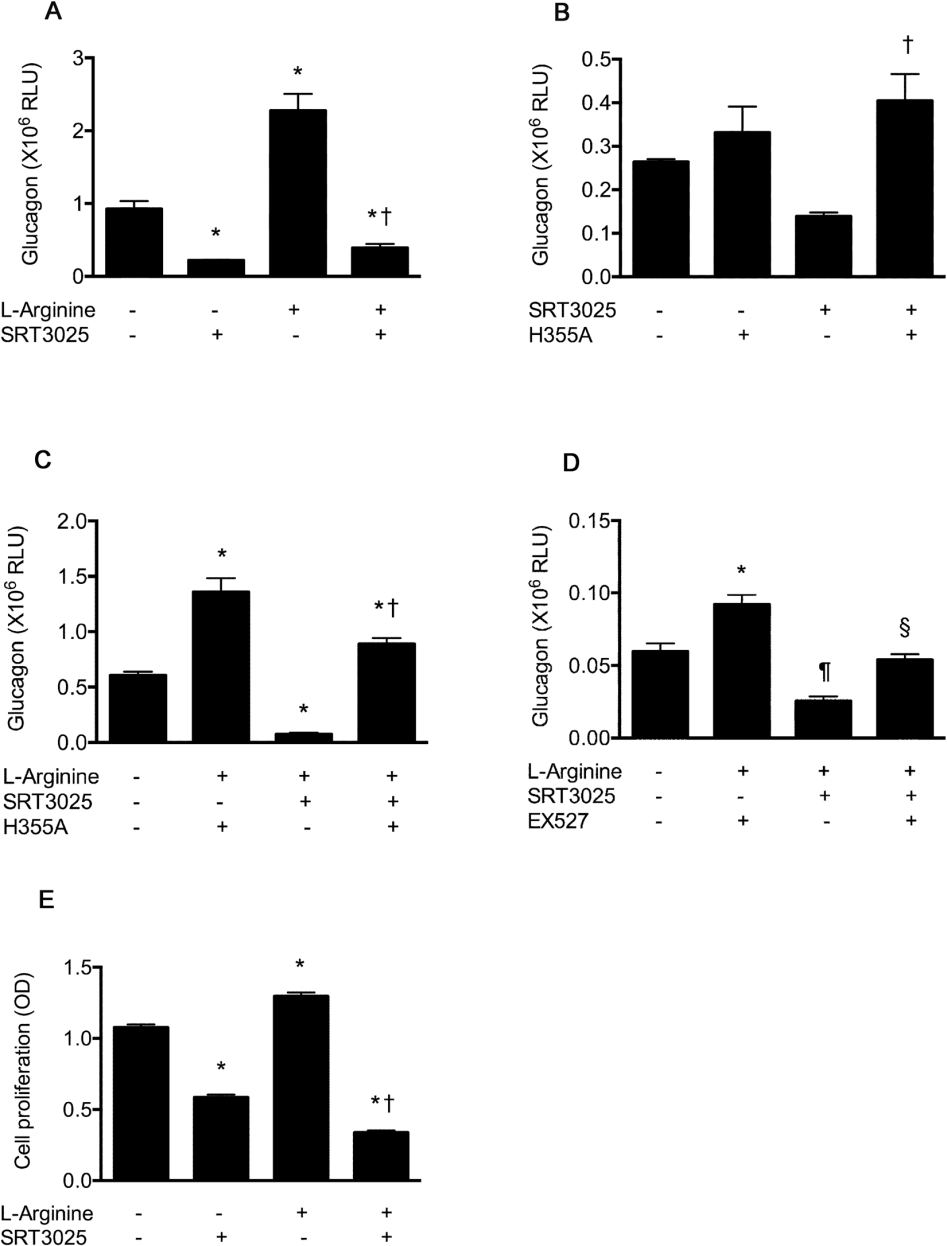
Cultured In-R1-G9 α cells were transfected with proglucagon (GLU)/Luciferase (LUC) reporter construct. SRT3025 reduced proglucagon reporter activity in the basal state and in response to l-arginine stimulation (**A**). Co-transfection with a catalytically inactive mutant Sirt1 (H355A) abrogated these effects in both the basal state (**B**) and in response to l-arginine (**C**) as did the SIRT1 inhibitor, EX527 (**D**). α cell proliferation was similarly reduced in the basal state and in response to l-arginine stimulation (**E**). N = 3/group. RLU, relative light units; OD, optical density. *p < 0.05 versus control; ^†^p < 0.05 versus arginine stimulation; ^‡^p < 0.05 versus SRT3025 treatment; ^§^p < 0.05 versus SRT3025 with arginine stimulation; ^¶^p < 0.05 versus EX527 with arginine-stimulation.

Having observed major changes in α cell mass and plasma glucagon with SRT3025, we next explored its effects on α cell secretion and proliferation in cultured cells. In both the presence and absence of l-arginine, SRT3025 substantially diminished glucagon reporter activity (Fig. 6). To confirm that these effects were the result of SRT3025’s effect on SIRT1, we repeated the above experiment after transfecting In-R1-G9 cells with a catalytically inactive SIRT1 construct (H355A). In these studies, H355A prevented the suppression of glucagon reporter activity by SRT3025, showing, in addition, augmentation of reporter activity in the presence of l-arginine. To further confirm that these effects were SIRT1-mediatied, In-R1-G9 cells were cultured in the presence of a selective SIRT1 inhibitor, EX527, that like H355A, prevented the SRT3025-induced diminution in glucagon reporter activity. α cell proliferation was similarly reduced by SRT3025 when incubated in a high glucose environment with and without l-arginine (Fig. 6).

### SRT3025 modulates islet cell fate transcription factor

**Figure 7:**
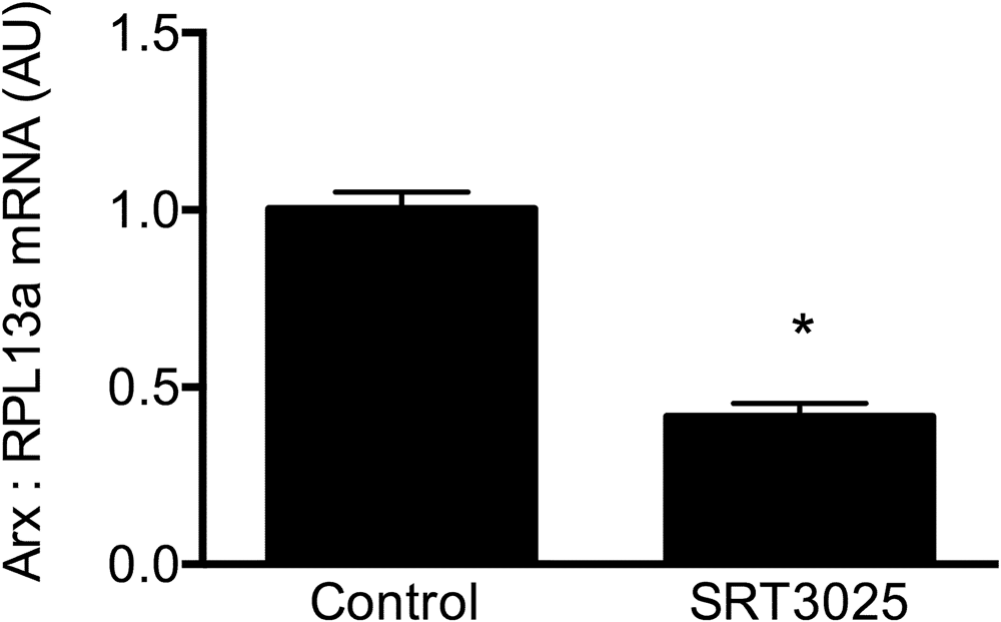
AXL mRNA in cultured α cells in response to SRT3025 as assessed by quantitative real-time PCR and expressed relative to untreated cells that were arbitrarily assigned a value of 1. *p < 0.05 vs. control.

Given the ability of SRT3025 to reduce glucagon expression in a SIRT1-dependent manner in α cells we next examined the effects of SIRT1 activation on the expression of factors linked to α and ß differentiation. After 48 hours, SRT3025 substantially reduced the expression of ARX, a transcription factor that is critical for α cell differentiation (Fig. 7). There were no significant differences in the expression of ß-cell differentiation factors such as PDX1 and insulin in the α cell line.

## Discussion

Studies of SIRT1 in Type 1 diabetes have to date been mostly confined to its potential role in human autoimmunity with the identification of a gene mutation that gives rise to a monogenic form of the disease^8,12^. Using a non-immune mediated streptozotocin model of type 1 diabetes, here we show that administration of an allosteric activator of SIRT1, SRT3025, led to substantial improvement in glycaemic control in the absence of any demonstrable effect on ß-cell mass, insulin secretion or insulin sensitivity. Rather, SRT3025 markedly attenuated α cell hyperplasia and hyperglucagonaemia.

In addition to its well-known endocrine effects, high intra-islet insulin inhibits glucagon secretion and α cell proliferation through paracrine mechanisms^13^. As such, the immunoselective destruction of ß-cells in patients with type 1 diabetes typically leads to a 2–3 fold expansion of α cell mass^10^. Consistent with previous reports, we also found that the near-complete ß-cell destruction with STZ is accompanied by increased islet α cell mass and elevated circulating concentrations of glucagon^10,14^. In contrast, islets from mice that had received SRT3025 showed a significant attenuation in α cell expansion along with a substantial suppression in the rise of plasma glucagon concentration in diabetic mice. Notably, SRT3025 had no demonstrable effect on the sparse number of insulin immunopositive ß cells nor on the commensurately low plasma insulin concentration.

Studies examining the role of hyperglucagonaemia in type 1 diabetes have yielded complex findings^9^. For instance, STZ-diabetic rodents develop near-complete ß-cell destruction and severe hyperglycaemia^10,15^ but if the STZ is administered on a glucagon receptor knockout

(*Gcgr*^*−/−*^) background, the mice remain normoglycaemic^16,17^. However, if diphtheria toxin is used, ß-cell destruction is complete and hyperglycaemia then ensues in these same (*Gcgr*^*−/−*^) mice^18,19^. Together, these data suggest, as detailed in a recent consensus statement, that in order for glucagon reduction to have a meaningful anti-hyperglycaemic effect, sufficient basal insulin also needs to be present^20^. As such, the clinical relevance of the current study’s findings in which the plasma insulin, though low, was nevertheless still present, as were sparse ß-cells, may be most applicable to those ~30% of patients with auto-immune destructive (type 1A) diabetes in whom numerous ß cells remain or in antibody negative (type 1B) disease in which insulin positive cells, though reduced in number, are still found in all islets^21^. Moreover, the markedly improved glycaemia with SRT3025 in association with reduction of hyperglucagonaemia is consistent with the findings of other studies that have used alternative strategies to attenuate glucagon action including immunoneutralization^22^ and anti-sense oligonucleotides^23^ as well as peptidic^24^ and non-peptidic small molecule antagonists^25^.

To elucidate the potential mechanisms that may account for the absence of α cell expansion and lower circulating glucagon concentrations in SRT3025-treated diabetic, we examined the effects of SIRT1 activation in cell culture. In cultured α cells, SRT3025 supressed glucagon reporter activity in the presence of high glucose, an effect that could be abolished by either overexpressing the catalytically-inactive SIRT1 mutant, H355A or the addition of the selective SIRT1 inhibitor, EX527. A similar, SRT3025-mediated reduction in α cell proliferation was also evident. Since these *in vitro* studies mirrored the findings of the *in vivo* setting, we next explored factors that might be responsible for the blunted α cells response, focussing on the determinants of α cell fate. Although incompletely understood, the transcription factor, ARX, is viewed as playing a key role in both α cell development and hyperplasia whereby its loss or diminution is linked to α cell deficiency and diminished α to ß cell conversion^26–30^. In the present study, we found that SRT3025 effectively reduced the expression of ARX in cultured α cells in association with reduced α cell proliferation and glucagon production. There was not, however, any evidence of their conversion to ß cells where transcription factors and markers of such differentiation such as PDX1, NKX6.1 or insulin^30^ were either absent or unchanged by SRT3025.

In contrast to its effects on α cells in diabetic mice, in the non-diabetic setting SRT3025 had its predominant effect on insulin-producing ß-cells. Not only was ß-cell mass reduced but trends towards reduced plasma insulin and increased insulin sensitivity were also seen in non-diabetic mice that had received SRT3025. For instance, expression of the rate-controlling enzyme of hepatic gluconeogenesis, PCK1 (PEPCK) was reduced in SRT3025-treated non-diabetic animals though, notably, blood glucose concentrations were unchanged. These findings are consistent with the published literature demonstrating the importance of SIRT1 in augmenting insulin sensitivity^7,31–33^.

Weight loss is a characteristic feature in rodent models of type 1 diabetes that reflects insensible energy loss through glucosuria as well as a catabolic state that is induced by insulin deficiency. This likely accounts for much of the weight difference between non-DM and diabetic mice that received SRT3025. However, a lower body weight was also noted in non-diabetic mice that received SRT3025 that could not be explained by changes in food intake. Similar reductions in body weight have been previously described in studies of SIRT1 activation in association with increased physical in activity, hypothesized to reflect changes in hypothalamic function^34–36^.

The current study has limitations. First and foremost, it is a study of an animal model not of type 1 diabetes in humans. Since SIRT1 activating compounds (STACs) are currently undergoing evaluation in clinical trials, we used a member of this drug class so that its findings may potentially be translatable to the clinical context. The precise mechanisms by which STACs exert their effects have become a matter of intense investigation^37^. While not direct activators of SIRT1^38^, STACs do still activate SIRT1, albeit indirectly, achieving their effects through allosteric modification of the enzyme^37,39,40^. Consistent with these findings, we showed, similarly, that the effects of SRT3025 on α cells could be blocked by overexpressing a catalytically-inactive SIRT mutant and by the SIRT1 inhibitor, EX527. However, examination of alpha cell function in the present study was undertaken in a cultured α cell line rather than from isolated intact islets obtained at termination.

In summary, administration of the SIRT1 activator, SRT3025, to mice with STZ-induced diabetes led to improved glycaemia with reductions in plasma glucagon and α cell hyperplasia without affecting either insulin secretion or ß-cell mass. Accordingly, this strategy may offer a new, α-cell centric approach to the treatment of certain patients with type 1 diabetes that complements the role of SIRT1 in preventing organ-specific autoimmunity^8^.

## Methods

### Animal experiments

One hundred and five, 6-week-old male CD1 mice were purchased from Charles River (Wilmington, MA). At 8 weeks of age, mice were randomised to receive a single dose of streptozotocin 150 mg/kg (Sigma, St. Louis, MO) or citrate buffer. Forty-eight hours later, when diabetes was confirmed by blood glucose >15 mmol/l, both diabetic and non-diabetic animals were randomized to receive either SRT3025, an allosteric activator of SIRT1^39,41,42^ milled in chow (3.18 g/kg), or regular chow. Of note, this compound has not been associated with significant toxicity in animal studies and although it did progress into clinical trials, dose-dependent prolongation of the QTc interval was noted in the latter with its further development now discontinued^43^.

Mice were housed in a temperature-controlled room (22 °C) with a 12 h:12 h light-dark cycle with free access to food and water at the St. Michael’s Hospital Animal Research Vivarium. At the end of the study, core body temperature was measured rectally by thermocouple probe (ATP Instrumentation Ltd., Leicestershire, UK).

After 20 weeks of treatment, animals were terminated at the end of a 12-hour dark (feeding) period when urine and fasting blood samples were collected, and multiple organs including the pancreas were harvested and either fixed in formalin or snap frozen and stored at −80 °C. All animal studies were approved by the St. Michael’s Hospital Animal Ethics Committee in accordance with the NIH Guide for the Care and Use of Laboratory Animals, Eighth edition (2011).

By the end of the study of the study, 20 weeks after receiving STZ, 9 diabetic animals had died, 4/24 in the vehicle-treated group and 5/29 in mice that had received SRT3025. There were no deaths in either of the non-diabetic groups.

### Metabolic studies and hormone measurements

Metabolic studies and hormone measurements were undertaken as previously described^44^. In brief, blood glucose concentrations were determined colorimetrically (Glucose Assay Kit, Eton Biosciences Inc., San Diego, CA). Blood glucose was measured after a 12 hour fast and during feeding at study end. Food intake was assessed by calculating food intake (g/mouse/day) over a 7-day period during the final week of the study. Insulin was measured at week 20 by ELISA (Crystal Chem, Downers Grove, IL) and serum glucagon was measured by RIA (Millipore, Burlington, MA, Cat. #GL-32K) at the Hormone Assay & Analytical Services Core at Vanderbilt University (Nashville, TN) in a random subset of 47 mice. Haemoglobin A_1c_ was measured on ethylenediamine tetra-acetic acid (EDTA) blood by kit assay (A_1c_Now + , Bayer, Sunnyvale, CA) in a random subset of 51 animals.

Insulin tolerance tests were performed during the last week of study on fasted animals by i.p. injection of human regular insulin (Novolin R, Novo Nordisk, Denmark) 0.75 U/kg body weight in 12 animals (n = 3/group), as previously described^4^, with blood glucose measurements at 0, 15, 30, 45, 60 and 120 minutes. The homeostasis model assessment method was used to determine insulin resistance (HOMA-IR) using the following equation: HOMA-IR = fasting insulin (µU/ml)** × **fasting glucose (mmol/l)/22.5^45^ where because of the near-undetectable insulin in STZ-diabetic mice, was calculated in 22 non-diabetic animals.

### Light microscopy

Pancreas immunohistochemistry was undertaken conducted in 28 randomly selected animals. In brief, pancreata were fixed in 10% neutral buffered formalin, pH 7.4 for 24 hours prior to routine processing and embedding in paraffin, sectioning and staining, as previously reported^46^ with antibodies against insulin (Dako, Glostrup, Denmark) and glucagon (Novocastra Laboratories, Newcastle upon Tyne, U.K.). Total islet area, islet size and total pancreas area were determined, as also previously described^46^. Stained sections were scanned using a ScanScope ImageScope system and analyzed with ImageScope version 9.0.19.1516 software (Aperio Technologies, Vista, CA).

### Quantitative PCR

The expression of enzymes, transporters and transcription factors associated with hepatic intermediary metabolism were assessed in the livers of 32 randomly selected mice that had been stored at −80 °C following their termination. These included major control-point enzymes of gluconeogenesis; phosphoenolpyruvate carboxykinase (PCK1, PEPCK) and glucose-6-phosphatase (G6Pase, G6PC); fat metabolism, CD36, and fatty acid synthase (FAS); along with transcription factors, Forkhead Box O1 and O6 (FoxO 1, 6).

mRNA expression of the α cell lineage transcription regulator, Aristaless Related Homeobox (Arx), was examined in In-R1-G9 cells after incubating them for 48 hours with or without SRT3025 (5 µmol/l).

Transcript abundance was assessed using SYBR green-based measurement and expressed relative to the housekeeping gene ribosomal protein L13a (RPL13a), as previously described^4^, using the predesigned sequence-specific primers (Table 2) and the QuantStudio 7 Flex Real-Time PCR System (Applied Biosystems, Foster City, CA) according to the manufacturer’s instructions. Experiments were performed in triplicate with data analyses undertaken using the Applied Biosystems Comparative CT method. The abundance of transcript among the different groups were compared with that in the vehicle-treated non-diabetic group that was arbitrarily assigned a value of 1 arbitrary unit (AU).

**Table 2:** List of primer sequences used for RT-PCR analysis in this study.

Target gene	Sequence (5′ → 3′)	
RPL13a	Sense	GCTCTCAAGGTTGTTCGGCTGA
	Antisense	AGATCTGCTTCTTCTTCCGATA
G6PC	Sense	GGAGGCTGGCATTGTAGATG
	Antisense	TCTACCTTGCTGCTCACTTTC
PCK1	Sense	TGATCTTGCCCTTGTGTTCTG
	Antisense	GTATCATCTTTGGTGGCCGTA
CD36	Sense	CCAGTCTCATTTAGCCACAGT
	Antisense	TGCAGGTCAACATATTGGTCA
FASN	Sense	ACTCCTGTAGGTTCTCTGACTC
	Antisense	GCTCCTCGCTTGTCGTC
FOXO1	Sense	GTCAAGACTACAACACACAGC
	Antisense	AAAACTATAAGGAGGGGTGAAGG
FOXO6	Sense	AGGATAAAGGCGACAGCAAC
	Antisense	CACCATGAACTCTTGCCAGT
AXL	Sense	CCTTATGCCGATCTACCATGAC
	Antisense	GTACCGTGTCCGAAAGTCC

### Cell culture

When unaccompanied by an increase in insulin, hyperglycaemia stimulates rather than suppresses glucagon secretion, exaggerating postprandial hyperglycaemia in untreated type 1 diabetes^9^. Accordingly, we sought to assess the effects of SRT3025 on glucagon gene expression in the high glucose (25 mmol/l) setting with and without the co-stimulatory effects of L-arginine, using the pancreatic α cell line, In-R1-G9, cultured as previously described^46^. Proglucagon transcriptional activity was assessed using a proglucagon (GLU)/Luciferase (LUC) reporter construct^47^. In brief, In-R1-G9 cells were plated (0.15 × 10^6^ cells/well) onto 24-well plates and after 24 hours were cotransfected with GLU-LUC and the normalizing vector RL-TK renilla luciferase along with empty vector. To confirm that the effects of SRT3025 were mediated by activating SIRT1 we transfected cells with a catalytically inactive mutant SIRT1 construct (Flag-SIRT1, H355A, Addgene) using Lipofectamine 2000, (LifeTechnologies) according to the manufacturer’s recommendation as previously reported^48^ with further confirmation also provided by co-incubating cells with the selective SIRT1 inhibitor, EX527 (30 µmol/l, Selleckchem, Houston, TX)^49^. After 16 hours, cells were serum starved for 3 hours, incubated with 25 mM glucose or arginine for 24 hours with or without SRT3025 (5 µmol/l) following which cells were lysed. Luciferase activity was determined using the Dual Luciferase Reporter Assay System kit (Promega) and a luminometer (Lumat 9507; Berthold). For each condition, treatments were performed in duplicates, and experiments were repeated at least three times. From each sample, firefly luciferase activity was normalized to the renilla luciferase activity of the same sample. Results were then expressed as fold changes compared with the mean firefly/renilla ratio of untreated controls.

For the assessment of cell proliferation, In-R1-G9 cells were serum starved, then incubated with glucose or L-arginine for 48 hours with or without SRT3025 (5 µmol/l) prior to cell counting by colorimetry (CellTiter 96^®^ AQ_ueous_ One Solution Reagent, Promega, Madison, WI).

### Statistics

Data are expressed as means ± SEM unless otherwise specified. As all data were normally distributed so that only parametric tests were used. For two group data sets analysis was done by unpaired t-test. One way ANOVA with with Fisher’s Protected Least Significant Difference *post hoc* testing for 3 or more independent groups. The data sets for the insulin tolerance test were analysed by 2-way ANOVA comparing the effects of time and treatment group assignment. All statistical analyses were performed using GraphPad Prism 6 for Mac OS X (GraphPad Software Inc., San Diego, CA). A p value of < 0.05 was regarded as statistically significant.

## Data Availability

All data generated during this study are included in this published article or are available from the corresponding author upon reasonable request.
